# Supplementary material for: The Binding of Different Substrate Molecules at the Docking Site and the Active Site of γ-Secretase Can Trigger Toxic Events in Sporadic and Familial Alzheimer’s Disease
Source: Int J Mol Sci. 2023 Jan 17;24(3):1835. doi: 10.3390/ijms24031835 (PMC9915333; doi:10.3390/ijms24031835)
Supplement: Supplementary file 1 [file ijms-24-01835-s001.zip › Svedruzic_supp_Gsec_dock_Ms_12_2022.pdf]

Online Supplement

Binding of different substrate molecules at the docking site and the active site of γ-secretase can trigger toxic events in sporadic and familial Alzheimer's disease

Željko M. Svedružić [1,2,4], Vesna Šendula Jengić [2], Lucija Ostojić [1,3]

[1] Laboratory for Biomolecular Structure and Function. Department of Biotechnology, University of Rijeka, 51000 Rijeka, Croatia.

[2] Laboratory for Medical Biochemistry, Psychiatric Hospital Rab, Kampor 224, 51280 Rab, Croatia

[3] current address: Department of Chemistry & Molecular Biology, Medicinaregatan 9 c, Box 462, 40530 Göteborg, Sweden

[4] Corresponding author: email: zeljko.svedruzic@uniri.hr

Table of Contents

1. Supplement video S1. Coarse-grained molecular dynamics studies of interactions between two free C99-βCTF-APP molecules in a cholesterol-lipid-bilayer 2
2. Supplement video S2. Coarse-grained molecular dynamics studies of interactions between of free C99-β-CTF-APP substrate (PDB:2LP1) and γ-secretase in complex with Aβ 1-49 substrate (PDB:6IYC) 2
3. Supplement video S3. All atom molecular dynamics studies of docking interactions between C-terminal section of C99-βCTF-APP substrate and cytosolic section of γ-secretase-Aβ49 complex. 2
4. Supplement video S4. Conformational changes in the active site tunnel for WT (right) and G384A FAD mutant (left) of presenilin 1 3
5. Supplement video S5. Coarse-grained molecular dynamics studies of interactions between the soluble part of human β-secretase (BACE1, PDB:4FGX) and γ-secretase in the absence of substrates (PDB:6IYC). 3
6. Supplement Figure S1. Coarse-grained MD studies of changes in C99-βCTF-APP structures in the cholesterol-lipid bilayer 5
7. Supplement Figure S2. Interaction sites in dimerization of two C99-βCTF-APP molecules in the cholesterol-lipid bilayer 5
8. Supplement Figure S3 (A-D). Cross-eyed stereoview view of conformational changes in γ-secretase complex upon closing of the nicastrin ectodomain 6
9. Supplement Figure S4 (A-C). Adaptive Poisson-Boltzmann Solver (APBS) protocols were used for calculation of isopotential surfaces (A), field gradient lines (B), and electric potentials mapped on the protein surface (C) 7
10. Supplement Figure S5 (A-C) Contact sites between γ-Secretase and its free C99-βCTF-APP substrate when the N-terminal end of the bound Aβ 1-49 substrate is buried below the surface of nicastrin ectodomain. 9
11. Supplement Figure S6 (A-C) Interaction sites between γ-secretase and free C99-βCTF-APP substrate when the N-terminal end of bound Aβ 1-49 substrate is exposed on the surface of nicastrin ectodomain. 11
12. Supplement Figure S7. The contact sites between intracellular part of presenilin subunit of γ-secretase and C terminal domain of free C99-βCTF-APP substrate. 12
13. Supplement Figure S8 (A-B). All-atom MD studies of distances between active site Asp257 and Asp385 in presenilin 1 13
14. Supplement Figure S9. The docking surface between BACE1 (PDB:4FGX) and ectodomain of nicastrin subunit of γ-secretase. 14
15. References 15

Supplement video S1. Coarse-grained molecular dynamics studies of interactions between two free C99-βCTF-APP molecules in a cholesterol-lipid-bilayer. Two C99-β-CTF-APP molecules are presented as CG structures [1]. The two proteins are shown as Connolly surfaces, with each protein colored in different shades as hydrophobic (white), positive (blue), negative (red), and polar not charged (green) [2]. The translucent spheres depict a cholesterol-lipid bilayer (methods). The colors highlight cholesterol (orange) in a mixture of POPC, POPA, POPE, POPS, POPI, and PSM molecules (cyan). Yellow-cyan CG spheres represent 150 mM potassium chloride ions [1].

MD calculations show 20 μsec of dynamic C99-β-CTF-APP structures that can be correlated with other structural studies [3-7]. The calculations started with fully extended soluble domains that were attached to the known transmembrane structure (PDB:2LP1, [3]), i.e. no secondary structure presumption for soluble parts (see methods). Intramolecular interactions start immediately at the start of calculations and lead to a dynamic compact structure for each soluble domain (supp. Fig S1). The two molecules gradually show dynamic transient contacts that come and break at different sites. The process is a result of a competition between protein-lipid and protein-protein interactions (supp. Fig S1). The two molecules form contacts as intermolecular interactions compete with intramolecular interactions.

The two C99-β-CTF-APP molecules gradually form tight complementary surfaces throughout the entire protein length. Dimerization is affected by the hinge point at Gly38-Gly39 sites (green), and by competition between charged and polar amino acids at the C-terminal and N-terminal ends.

The first part of the video is focused on steps that show dimer formation, the second part is focused on dimer stability and structure. Contact sites are listed in supp. Fig S2. The transmembrane helix is hydrophobic (white) with notable polar sites at Thr 43 and Thr 48 sites (green). For clarity cholesterol-lipid bilayer and potassium chloride ions are shown as static spheres that have been sliced in the plane of the protein [2]. The static dots were used to represent one out of every 50 water molecules [2].

Supplement video S2. Coarse-grained molecular dynamics studies of interactions between of free C99-β-CTF-APP substrate (PDB:2LP1, [3]) and γ-secretase in complex with Aβ 1-49 substrate (PDB:6IYC, [8]). CG structures [1] of all proteins are depicted as partially transparent Connolly surfaces to make the interaction sites visible [2]. γ-Secretase complex shows nicastrin (pink), presenilin 1 (white), Aph1 (yellow), and Pen2 (hidden). C99-βCTF-APP and Aβ 1-49 substrates are colored as hydrophobic (white), negative (red), positive (blue), and polar not charged (green). The cholesterol-lipid bilayer (methods) is depicted with translucent CG spheres [1] to show cholesterol (orange) and other lipids (cyan). Multiscale MD studies can describe 10 μsec of diffusion in the cholesterol-lipid bilayer that can be driven by the complementary electric fields (supp. Fig. S4). The corresponding changes in protein structures lead to a gradual buildup of docking interactions.

The video shows specific conformations where the bound Aβ 1-49 substrate has its N-terminal maximally shielded by the nicastrin ectodomain (Fig. S3 in the main text). In those conditions, the closed nicastrin ectodomain can maximally block contacts between the N-terminal domains of the two substrates. Interestingly, the C-terminal domain of free C99-βCTF-APP substrate can extend towards cytosolic sections of TM6, TM6a, TM7, and the endoproteolytic site on presenilin 1. These are the most dynamic sites in presenilin structure [8,9], which control processive cleavages in Aβ production. These sites can be affected by the drugs and the FAD mutations [8-11]. Contact sites between γ-secretase and second C99-βCTF-APP substrate are listed in supp. Fig. S5.

Supplement video S3. All atom molecular dynamics studies of docking interactions between C-terminal section of C99-βCTF-APP substrate and cytosolic section of γ-secretase-Aβ49 complex [8]. The video is derived from video S2, using conversion from CG-MD to AA-MD structures [12]. The aim is to describe to atomic details dynamic docking interactions between the C-terminal domain of the free substrate and the cytosolic end of presenilin 1 (molecular timeframe 300 nanoseconds). For clarity, this video is shows only docked C99-βCTF-APP substrate, bound Aβ 1-49 substrate, and the presenilin subunit (N-terminal gray, C-terminal pink [8]). The backbone models are used to depict protein conformers, while the transparent Connolly surfaces are used to depict protein-protein contacts [2]. The backbone of C99-βCTF-APP and

bound Aβ 1-49 substrate are colored as hydrophobic (white), negative (red), positive (blue), and polar not charged (green). The red blobs represent the active sits AspH257 and Asp385 [8]. The video shows how the substrate C-terminal domain can dock with its full length to the most dynamic catalytic parts on presenilin structure, while the N-terminal domains of the two substrates is nicely separated due to the closing of nicastrin ectodomain (compare with video S2). Contact sites are listed in supp. Fig. S7. The video shows that the substrate C-terminal domain can affect the most dynamic presenilin sites. The same sites can get affected by the drugs and the FAD mutations [8-11].

Supplement video S4. Conformational changes in the active site tunnel for WT (right) and G384A FAD mutant (left) of presenilin 1 [8]. Multiscale MD studies were used to study conformational changes in the active site tunnel of WT and G384A FAD mutant of γ-secretase. We show some representative all atom data that correspond to 300 nanoseconds on molecular timescale. γ-Secretase structures are shown as ribbons with a focus on the presenilin subunits (cyan). The red beads represent the negative active sites Asp257 and Asp385 [8]. The blue beads represent some of the most dynamic positive amino acids at the cytosolic end of the active site tunnel (Lys 265, Arg269 Arg 278 on TM6a, Lys 429-430 at TM8 [8]). The small piece of yellow ribbon represents PAL motif, which can be affected by drugs (residues ProAlaLeu 433-435, [10,11]). The cholesterol-lipid bilayer, solvent, and KCl ions are not show for clarity (methods). With the G384A mutant, the negative active site Asp385 can make direct contact with the positive Arg269. With WT protein, the negative active site aspartates and the surrounding positive residues attract, but always remain clearly separated. The observed differences between the WT and G384A mutant can be traced to differences in width of the active site tunnel due to slightly bigger Ala384 residue. The Arg269-Asp385 contacts can make G384A mutant catalytically less efficient than WT enzyme ([13] and supp. Fig. S8B). With WT protein, the positive residues remain clustered at the end of the active site tunnel. The clustering leads to repulsion that can force the active site tunnel to stretch open at the end of calculations. In sum, we find that G384A FAD mutant and the second substrate can affect the same dynamic structures in the active site tunnel (compare supp. videos S3-S4). We also found that the same dynamic structures can get affected by drugs that can facilitate cognitive decline, such as semagacestat and avagacestat [11].

Supplement video S5. Coarse-grained molecular dynamics studies of interactions between the soluble part of human β-secretase (BACE1, PDB:4FGX, [14]) and γ-secretase in the absence of substrates (PDB:6IYC, [8]). CG structures [1] of all proteins are depicted as ribbon models [2], while the cholesterol-lipid bilayer is shown for clarity as a layer of static dots [1]. γ-Secretase complex shows nicastrin (pink), presenilin 1 (white), Aph1 (yellow), and Pen2 (orange). Highlighted are Asp257 and Asp385 in the active site (red beads). BACE1 (green) is shown with highlighted Asp 93 and Asp 289 in the active site (red beads). Ile447 (black beads) is highlighted to mark the position of the start of its transmembrane helix at the C-terminal end.

The video shows how multiscale MD studies can describe diffusion and initial contacts between the proteins. Initial protein-protein contacts result in conformational changes that lead to a gradual buildup of docking interactions [15]. The soluble BACE1 structure becomes anchored to the membrane surface by its interaction with γ-secretase. The anchoring can position C-terminal end of BACE1 at the membrane surface in the position where its transmembrane helix is expected to start. Interaction between BACE1 and nicastrin ectodomain is forcing the nicastrin ectodomain in its open position. Opposite movements in nicastrin ectodomain can be observed with isolated γ-secretase (video S2). The observed interactions are suggesting that substrate channeling can control the exchange of C99-βCTF-APP substrate between β-secretase and γ- secretase, and thus related pathogenic and pharmacologic events [16]. Different conformers from CG calculations can be used for conversion from coarse-grained to all-atom structures [12]. The prepared all atom structures can be used to calculate the contact surfaces to atomic details (Fig. S8 A-B, and supp. Fig. S9).

Molecular dynamics studies of secondary structure of a single C99-βCTF-APP

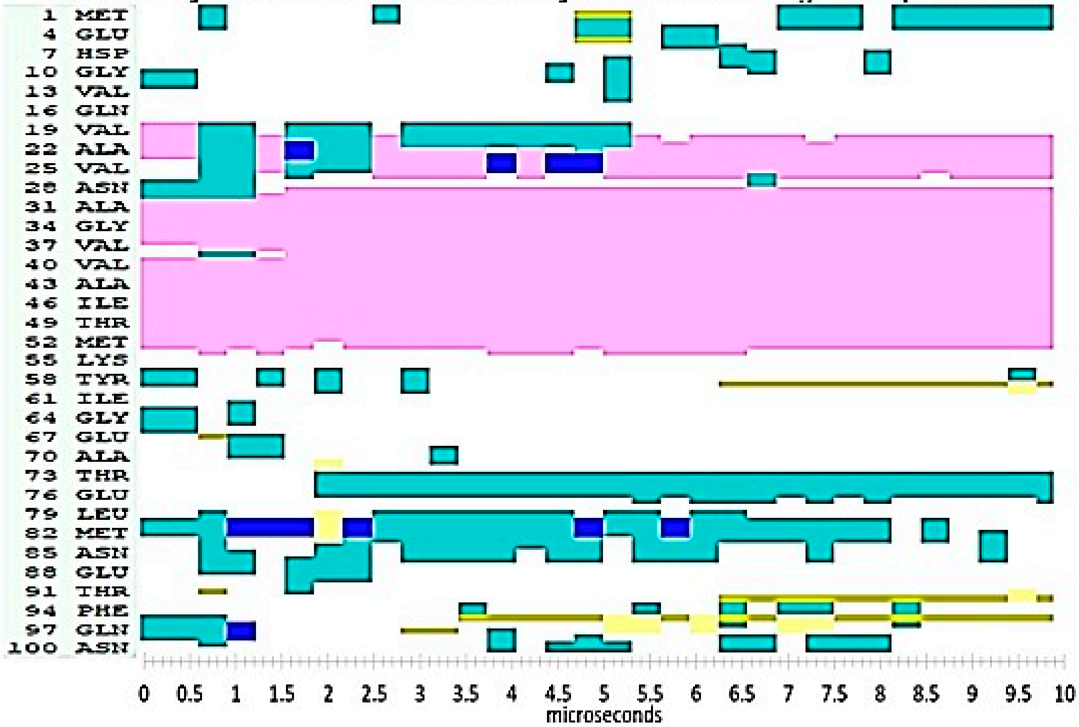

pink: helix/ blue: 3-10 helix / cyan: β-sheet/
white: coil /yellow: extended coil structure/ dark yellow: isolated bridge

C99-βCTF-APP - lipid interactions

N terminal blue, C terminal black

donor	acceptor	occupancy
POPE732-Main	ASP69-Side	43.75%
SER80-Main	POPC587-Side	37.50%
LYS54-Side	POPE728-Side	37.50%
POPE728-Main	GLU76-Side	28.13%
SER80-Side	POPC587-Side	25.00%
LYS54-Side	POPE730-Side	21.88%
HSP14-Side	POPC511-Side	21.88%
POPE730-Main	GLU76-Side	21.88%
GLN57-Side	POPE728-Side	18.75%
GLN16-Main	POPC512-Side	18.75%
HSP15-Side	POPC511-Side	18.75%
HSP15-Main	POPC511-Side	18.75%
GLN16-Side	POPC512-Side	18.75%
POPE728-Main	GLU75-Side	18.75%
GLN83-Side	POPC588-Side	15.63%
LYS55-Side	POPE731-Side	15.63%
GLN16-Side	POPC511-Side	15.63%
GLN57-Main	POPE730-Side	15.63%
LYS17-Side	POPC511-Side	12.50%
LYS55-Side	POPE729-Side	12.50%
POPE689-Main	ASP8-Side	12.50%
LYS81-Side	POPC587-Side	12.50%

C99-βCTF-APP – protein interactions

N terminal: extracellular

donor	acceptor	occupancy
VAL25-Main	PHE21-Main	43.75%
MET1-Main	GLU4-Side	43.75%
ALA22-Main	VAL19-Main	37.50%
SER27-Main	GLU23-Main	31.25%
SER27-Side	GLU23-Main	31.25%
ARG6-Side	GLU4-Side	25.00%
ARG6-Side	ARG6-Main	18.75%
HSD15-Side	GLU12-Side	18.75%
GLY26-Main	ALA22-Main	18.75%
VAL25-Side	PHE21-Main	18.75%
VAL25-Main	ALA22-Main	12.50%
VAL13-Main	TYR11-Main	12.50%
HSD7-Side	GLU4-Side	12.50%
HSD7-Side	ASP8-Side	12.50%

C99-βCTF-APP – protein interactions

C terminal intracellular

donor	acceptor	occupancy
LEU79-Main	VAL72-Main	87.50%
LYS93-Main	TYR58-Main	50.00%
ARG77-Side	GLU75-Side	25.00%
THR73-Side	ALA71-Main	25.00%
SER60-Main	THR91-Main	25.00%
GLY86-Main	GLN84-Side	18.75%
GLN83-Main	SER80-Main	18.75%
HSD78-Side	THR73-Side	18.75%
HSD63-Side	GLU96-Side	12.50%
TYR87-Side	GLN83-Side	12.50%
MET82-Main	LEU79-Main	12.50%
ASN85-Side	ASP69-Side	12.50%
ARG77-Side	ASN89-Side	12.50%
GLN97-Main	GLU96-Side	12.50%
TYR87-Side	HSD78-Main	12.50%
GLN83-Side	LYS81-Main	12.50%

Supplement Figure S1. Coarse-grained MD studies of changes in C99-βCTF-APP structures in the cholesterol-lipid bilayer**.** Coarse-grained MD studies can calculate conformers of C99-βCTF-APP molecules that represent 10 to 20 microseconds of molecular events [1]. Calculated coarse-grained structures can be converted to all-atom structures to describe to atomic details the conformational changes that take place within 0.5 microseconds [12]. The corresponding changes in secondary structure can be depicted using 2D plots [2]. Selected conformers were also used in all-atom MD studies to describe the competing interactions that influence C99-βCTF-APP structures as indicated in tables (300 nanoseconds of molecular events). The tables show all H-bonds that can be observed in the related all-atom calculations. The first table shows a list of all interactions between protein and polar lipid heads, for the extracellular side (blue), and for the intracellular side (black). The next two tables show list of interactions within the protein. The listed numbers give percentage of MD time in which related H-bond can be observed. The higher occupancy values correlate with the higher stability of interaction and the higher energy of interaction. The listed residues can form H-bonds between their main chains and side chains.

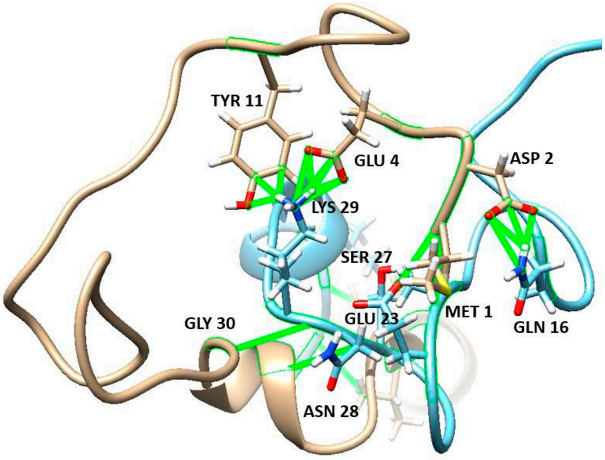

first C99-βCTF-APP	second C99-βCTF-APP	% occupancy
LYS 29 - Side	GLU 4 - Side	61
GLN 16 - Side	ASP 2 -Side	47
GLU 23 - Side	MET 1 - Main	45
LYS 29 - Side	TYR 11- Side	37
LYS 55 - Side	GLU 88 - Side	28
ASN 28 -Side	GLY 30 - Main	20
THR 49 - Side	TYR 87- Side	16

Supplement Figure S2. Interaction sites in dimerization of two C99-βCTF-APP molecules in the cholesterol- lipid bilayer**.** Coarse-grained MD calculations can map the conformers of C99-βCTF-APP molecules when two molecules are positioned together in cholesterol-lipid bilayer (video S1). Here we convert coarse-grained structures to all-atom structures to describe the interaction sites for selected conformers to atomic details [12]. Formation of C99-βCTF-APP dimers appears to be primarily driven by interaction between charged and polar amino acids on the extracellular N-terminal domain. The tables show all H-bonds that can be observed in the related all-atom calculations (300 nanoseconds of molecular events). The listed numbers give a percentage of MD time in which related H-bond can be observed. The higher occupancy values correlate with the higher stability of interaction and the higher energy of interaction. The listed residues can form H-bonds between their main chains and side chains.

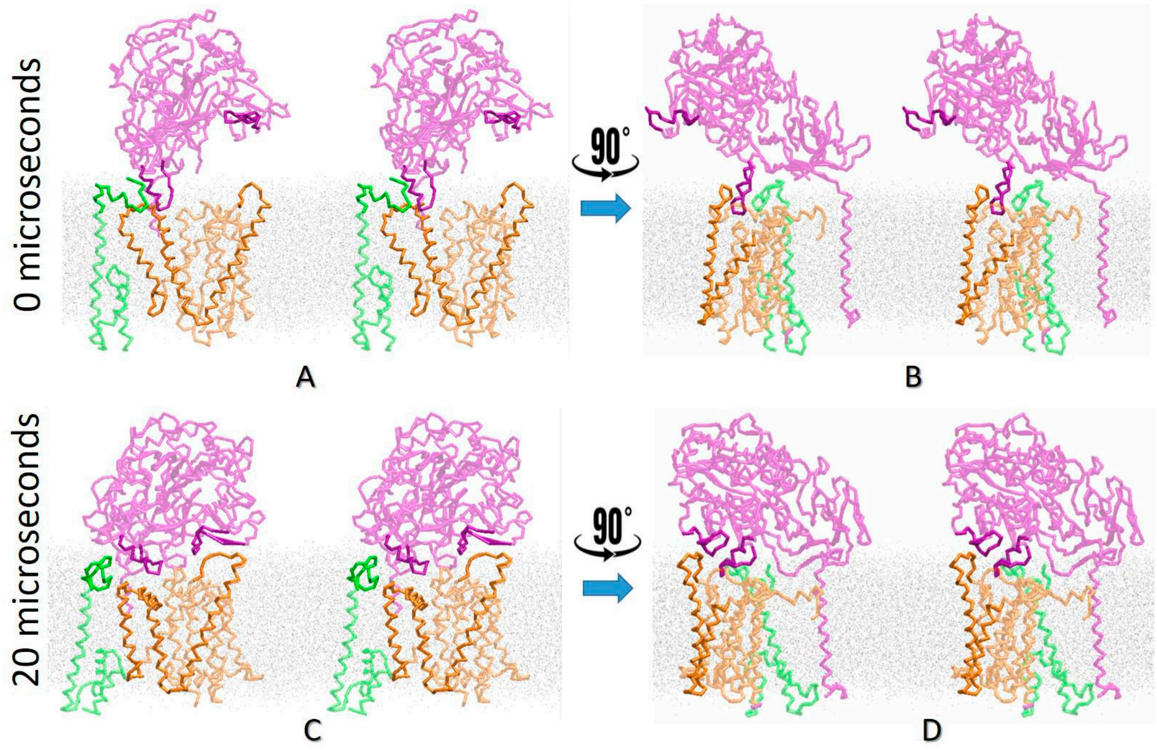

Supplement Figure S3 (A-D). Cross-eyed stereoview view of conformational changes in γ-secretase complex upon closing of the nicastrin ectodomain**.** The models show nicastrin (purple), psen1 (orange), psen2 (green), and membrane (gray dots), while for clarity the Aph1 subunit is not shown. The contact points between different subunits that drive the closing of the complex are highlighted. Silver dots represents cholesterol-lipid-bilayer.

(A-B) EM structures show γ-secretase with the nicastrin ectodomain open ([8] PDB:6IYC).

(**C-D**) Coarse-grained molecular dynamics calculations (20 microseconds) showed that the nicastrin ectodomain can close within the first several microseconds as reported in the earlier studies [17-19]. The closing is driven by the interaction between flexible loops on nicastrin and links TM1 and TM2 on presenilin 1 (nicastrin a.a. 555-572, presenilin 1 a.a. 111-123). The interaction between the two loops drives the breakdown of interaction between nicastrin and psen2 (helix a.a. 225-245 on nicastrin and a.a. 81 to 101 on presenilin 2). When psen2 is displaced, the nicastrin forms contact with TM3 (a.a. 187-195). Those contacts can displace TM4 and bend the TM3 by as much as 65 degrees in the position of Phe rich sequence that starts with the Phe175. The presented interactions drive the repositioning of TM2-TM3 relative to TM4 and TM6a (C-D), and exposure of otherwise buried TM6 and TM6a (D). The closing of the nicastrin ectodomain also results in its internal twist by 27 degrees, which results in a compact structure that cannot protrude beyond the presenilin structure (compare B and D).

A

B

C

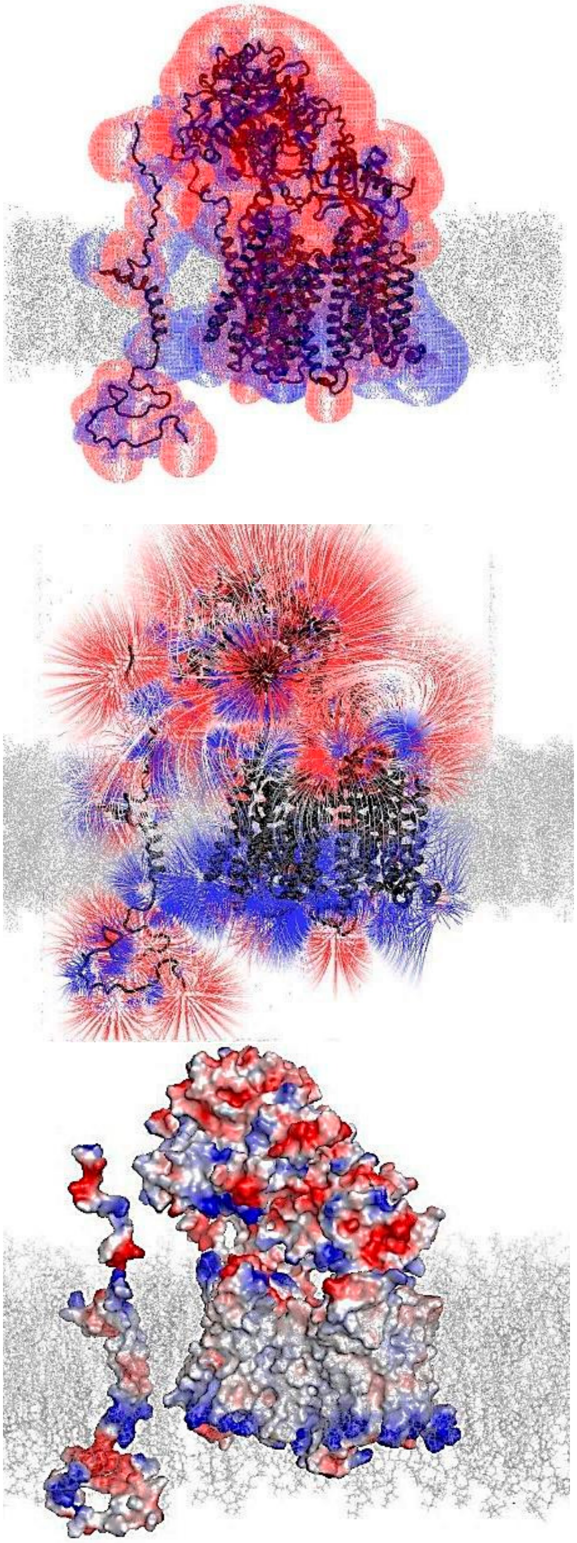

Supplement Figure S4 (A-C). Adaptive Poisson-Boltzmann Solver (APBS, [20]) protocols were used for calculation of isopotential surfaces (A), field gradient lines (B), and electric potentials mapped on the protein surface (C).

Protein are shown as a backbone ribbon, or as Connolly surface, while the silver dots represent cholesterol-lipid-bilayer (methods) [2].

(A) Blue and red dots represent positive and negative isopotential surfaces that are mapped in the space around the protein (scale 1.0 to -1.0 k_BT/e). γ-Secretase complex forms predominantly negative ispotential surfaces at the extracellular nicastrin site and predominantly positive isopotential surfaces around TM2, TM3, TM6 and TM6a, intracellular presenilin sites. The free C99-βCTF-APP substrate can form complementary electric fields.

(B) Blue and red lines represent gradient lines from positive to negative sites in the space around the proteins (scale 0.5 to -0.5 k_BT/e). γ-Secretase complex forms predominantly negative field at the extracellular nicastrin site and predominantly positive filed around intracellular TM2, TM3, TM6, TM6a, and TM7 sites on presenilin. The free C99-βCTF-APP substrate can form complementary electric fields that can drive the initial contacts.

(C) Blue and red patches represent positive and negative electric fields that are mapped on the Connolly surface (scale -4.0 to 4.0 k_BT/e). The figure shows that after the initial attraction, the buildup in of docking interactions in MD studies depends on conformational changes that can form complementary electric field patches on the surface of the two proteins.

Contact sites between nicastrine ectodomain and N-terminal of free C99-βCTF-APP substrate

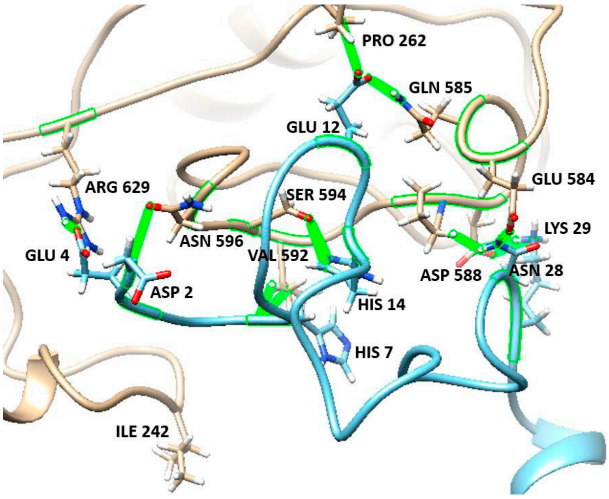

nicastrine	C99-βCTF-APP	% occupancy
ASP 588 - Side	LYS 29 - Side	83
GLU 584 - Side	ASN 28 -Side	61
GLN 585 - Side	GLU 12 - Side	54
SER 594 - Side	HIS 14 - Main	32
ARG 629 - Side	GLU 4 - Side	25
VAL 592 - Main	HIS 7 - Main	18
PRO 589 - Main	ASN 28 - Main	14

A

Contact sites between nicastrine ectodomain and N terminal of Aβ 1-49 bound in the active site

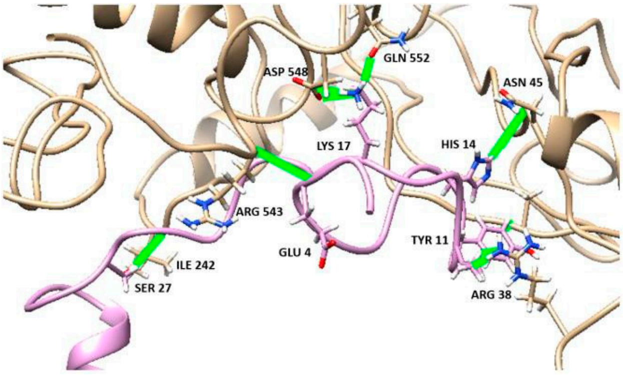

nicastrin	Aβ 1-49	% occupancy
ASP 548 -Side	LYS 17 -Side	88
GLN 552 -Side	LYS 17 -Side	72
ALA 658 -Main	TYR 11 - Side	48
ASN 45 - Main	HIS 14 - Side	42
ARG 38 - Side	VAL 13 - Main	41
Ile 242 - Main	SER 27 - Side	32

B

Contact sites between N terminals of Aβ 1-49 bound and docked C99-βCTF-APP substrate

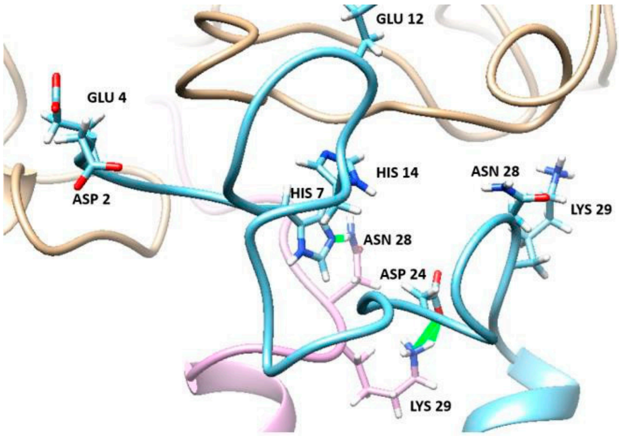

Aβ 1-49	C99-βCTF-APP	% occupancy
LYS29 - Side	ASP 24 - Side	17
LYS29 - Side	HIS7 - Side	14
ASN28 - Side	ASP24 - Side	12

C

Supplement Figure S5 (A-C) Contact sites between γ-Secretase and its free C99-βCTF-APP substrate when the N-terminal end of the bound Aβ 1-49 substrate is buried below the surface of nicastrin ectodomain. Coarse-grained MD studies can calculate different conformers and possible docking interactions between γ-secretase and its free C99-βCTF-APP substrates (supp. videos S2-S3). Here we convert coarse-grained structures to all-atom structures to describe the interaction sites for selected conformers to atomic details (Fig. 3, main text) [12]. The protein-protein complex shown in figure 3 was sliced at the interaction plane to expose buried interaction sites. The green lines represent H bonds between the nicastrin (gold) subunit of γ-secretase and the two substrates; C99-βCTF-APP substrate (cyan) bound at the docking sites, and Aβ 1-49 substrate (magenta) bound in the active site tunnel. The H-bond selection criteria are distances of less than 3.5 Å and angle smaller than ± 25 degrees (methods). The tables show all H-bonds that can be observed in the related all-atom calculations (300 nanoseconds molecular time). The listed numbers give a percentage of MD time in which related H-bond can be observed. These values are directly proportional to the stability of interaction. The higher occupancy values correlate with the higher stability of interaction and the higher energy of interaction. The listed residues can form H-bonds between their main chains and side chains.

Contact sites between nicastrine ectodomain and N-terminal of docked C99-βCTF-APP

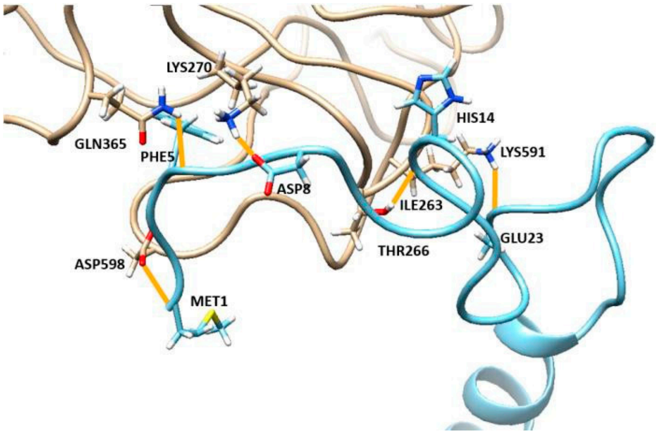

nicastrine	C99-βCTF-APP	% occupancy
LYS 591-Side	GLU 23-Side	59
ILE 263-Side	HIS 14-Side	43
LYS 270-Side	ASP 8-Side	31
ASP 598-Side	MET 1-Main	26
ASN 596-Side	PHE 5-Main	22
THR 266-Side	GLY 10-Main	18
THR 266-Side	His 14-Side	14
GLY 267-Main	His 7-Main	11

A

Contact sites between nicastrine ectodomain and N terminal of Aβ 1-49 bound in the active site

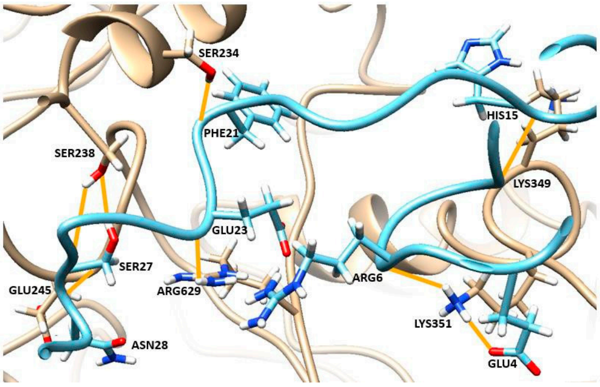

nicastrine	Aβ 1-49	% occupancy
LYS 351- Side	GLU 4 - Side	47
GLU 245 - Side	ASN 28 - Side	41
SER 238-Main	GLY 26 -Main	33
LYS 351 -Side	ARG 6 -Side	26
TYR 345 - Side	HIS 15 - Side	24
ARG 629 - Side	GLU 23 - Side	21
LYS 349 - Side	HIS 15 - Side	18
ARG 342 -Side	HIS 8 - Side	14
SER 234 - Main	ALA 22 - Main	12

B

Contact sites between intracellular domain of presenilin and C-terminal of docked C99-βCTF-APP

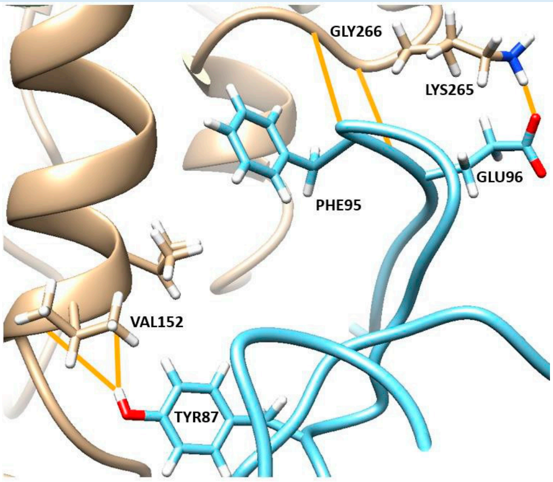

presenilin	C99-βCTF-APP	% occupancy
LYS 265 - Side	GLU 96 - Side	84
GLY 266 - Main	PHE 95 - Main	63
VAL 152 - Main	TYR 87 - Main	46

C

Supplement Figure S6 (A-C) Interaction sites between γ-secretase and free C99-βCTF-APP substrate when the N-terminal end of bound Aβ 1-49 substrate is exposed on the surface of nicastrin ectodomain. Coarse- grained MD studies can calculate different conformers and possible docking interactions between γ- secretase and its free C99-βCTF-APP substrates (Fig. 4, main text). Here we convert coarse-grained structures to all-atom structures to describe the interaction sites for selected conformers to atomic details [12]. The protein-protein complex shown in figure 4 was sliced at the interaction plane to expose buried interaction sites. The orange lines depict H bonds between the nicastrin (gold) or presenilin (gold) subunits of γ-secretase and its docked C99-βCTF-APP substrates (cyan). The H-bond selection criteria are distances of less than 3.5 Å and angle smaller than ± 25 degrees (methods). The table gives a complete list of all H-bonds that can be observed in the related all-atom calculations (300 nanoseconds molecular time). The listed numbers give a percentage of time in which related H-bond can be observed. These values are directly proportional to the stability of interaction and the energy of interaction. The higher percentage occupancy values correlate with the higher stability of interaction and the higher energy of interaction. The listed residues can form H-bonds between their main chains and side chains.

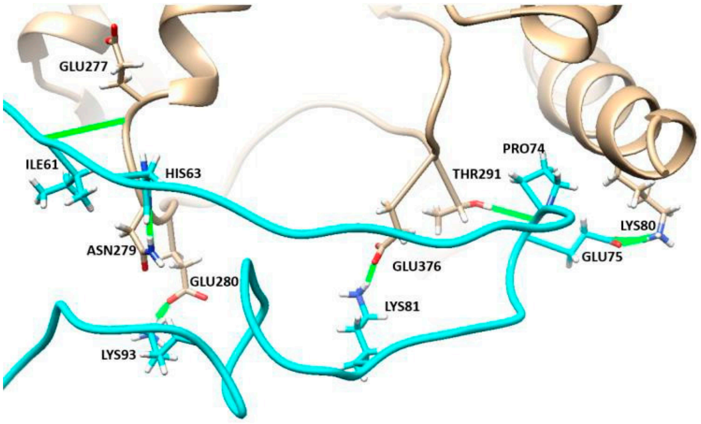

presenilin	C99-βCTF-APP	% occupancy
LYS80-Side	GLU75-Side	65
GLU376-Side	LYS81-Side	57
ARG377-Side	ASP69-Side	55
GLU280-Side	LYS93-Side	53
GLU277-Side	TYR58-Side	27
ASN279-Side	HIS63-Side	20
ASN279-Side	GLU96-Main	16
THR291-Side	PRO74-Main	14
GLU277-Main	ILE61-Main	12

Supplement Figure S7. The contact sites between intracellular part of presenilin subunit of γ-secretase and C terminal domain of free C99-βCTF-APP substrate. Coarse-grained MD studies have been used to calculate different orientations and different conformers that can support maximal docking interactions between γ-secretase and its free C99-βCTF-APP substrates (video S2). Here we convert coarse-grained structures for selected conformers (Fig. 7, main text) to all-atom structures to describe interaction sites to atomic details [12]. The protein-protein complex shown in figure 7A (main text) was sliced at the interaction plane to expose buried interaction sites. The green lines depict H bonds between intracellular parts of the presenilin subunit (gold) and C99-βCTF-APP substrate (cyan) bound at the docking sites. The H-bond selection criteria are distances of less than 3.5 Å and angle smaller than ± 25 degrees (methods). The table shows all H-bonds that can be observed in the related all-atom calculations (300 nanoseconds molecular time). The listed numbers give a percentage of MD time in which related H-bond can be observed (supp. video S3). These values are directly proportional to stability of interaction. The higher percentage occupancy values correlate with the higher energy of interaction. The listed residues can form H-bonds through their main chain or side chain atoms.

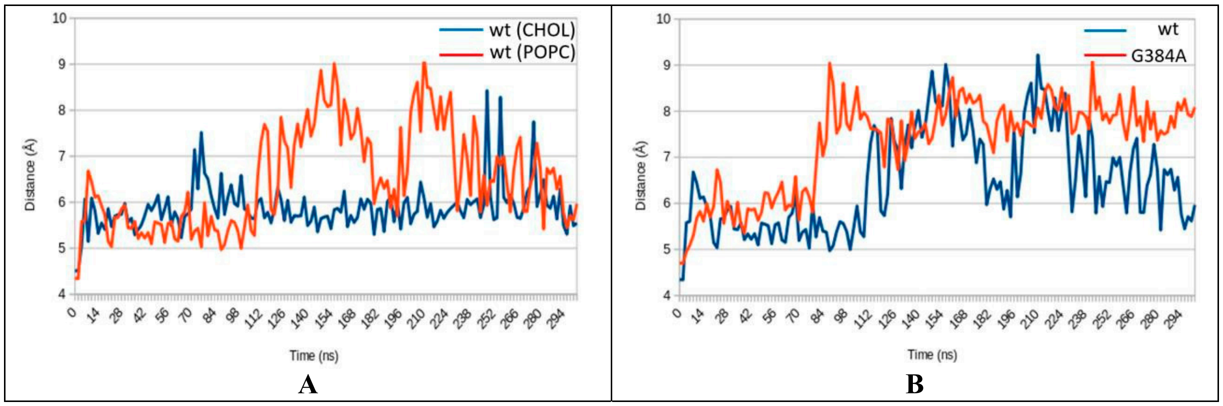

A **B**

Supplement Figure S8 (A-B). All-atom MD studies of distances between active site Asp257 and Asp385 in presenilin 1**.** The catalytic relevance of different presenilin structures in MD studies can be analyzed by following distances between γ-carbon atoms for the active site Asp257 and Asp385 [21-23]. The distance and angle between active site aspartates can affect their pKa values and thus catalytic efficiency. We use changes in distance between γ-carbon on its active site Asp257 and Asp385 to follow possible changes in catalytic efficiency of related presenilin structures [21]. The pKa for specific distances have been calculated and compared to the experimental pKa values [22,23].

(**A**) The distance between γ-carbon on AspH257 and Asp385 can be affected by the lipid bilayer [24,25]. The bigger distance in POPC membranes can be attributed to higher mobility for all amino acids in proteins that are embedded in POPC bilayers. The distance between γ-carbon on the active site aspartates in cholesterol-lipid-bilayer in our studies is comparable to similar studies in the past [21].

(**B**) FAD mutations can affect presenilin structure, especially the active site tunnel, by disrupting the optimal catalytic distance and orientation between γ-carbon atoms on Asp257 and Asp385 (video S4).

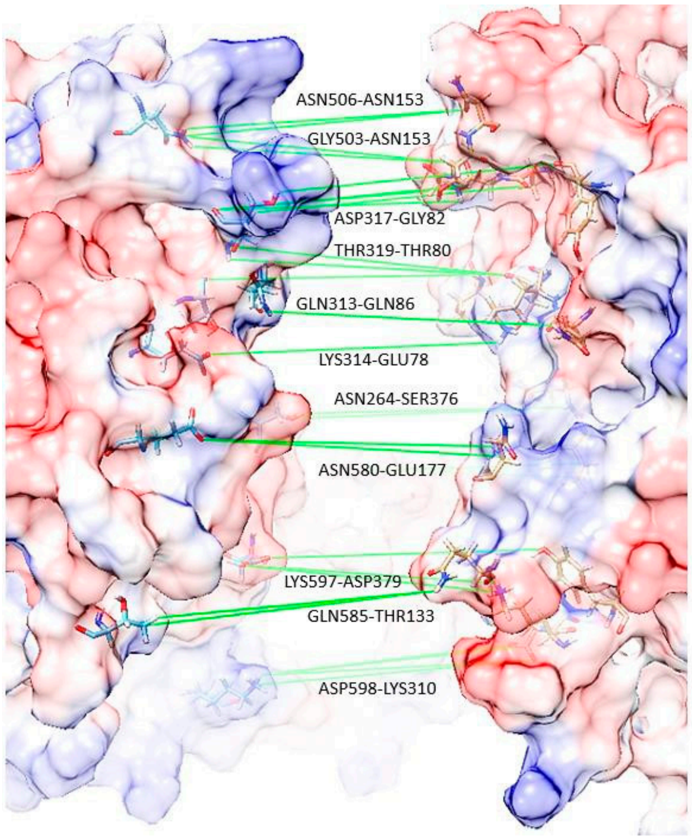

BACE1: PDB 4FGX	NICASTRINE	% occupancy
SER376	ASN264	74
TYR113	GLU310	42
ASP379	LYS597	33
TYR113	TYR499	29
GLU78	LYS314	25
ARG115	THR510	25
SER83	ALA502	25
GLU78	LYS314	24
TYR113	GLN512	24
SER59	THR319	23
LYS310	ASP598	23
SER174	THR582	22
SER376	ILE263	22
ASP379	TYR602	18
SER83	THR505	16
GLN86	GLU310	15
THR80	THR319	14
ASP372	ASN264	14
SER83	ALA502	13
GLU78	THR577	13
LYS310	ASP598	13

Supplement Figure S9. The docking surface between BACE1 (PDB:4FGX, [14]) and ectodomain of nicastrin subunit of γ-secretase. Coarse-grained MD studies can calculate how different orientations and different conformers can affect possible docking interactions between γ-secretase and human BACE1 (video S5). Here we describe to atomic details docking surfaces by converting coarse-grained structures to all-atom structures [12] for selected conformers (Fig 8A, main text). The resulting protein-protein complex is shown split apart and sliced at the interaction plane to expose the buried interaction sites. Protein surfaces are shown transparent, and colored according to their surface electric potential [20]. The presented figure shows that the two proteins can form a large and dynamic complementary surface. The green lines depict dynamic H bonds between intracellular parts of BACE1 (left) and nicastrin ectodomain (right). The H bonds selection criteria are distances of less than 3.5 Å and angles smaller than ± 25 degrees (methods). The table shows all H-bonds that can be observed in the related all-atom calculations (300 nanoseconds of molecular events). The listed numbers give percentage of MD time in which related H-bond can be observed (supp. video S5). These values are directly proportional to stability of interaction at that site. The higher percentage occupancy values correlate with the higher energy of interaction.

References:

1. Arnarez, C.; Uusitalo, J.J.; Masman, M.F.; Ingólfsson, H.I.; de Jong, D.H.; Melo, M.N.; Periole, X.; de Vries, A.H.; Marrink, S.J. Dry Martini, a coarse-grained force field for lipid membrane simulations with implicit solvent. *J Chem Theory Comput* **2015**, *11*, 260-275, doi:10.1021/ct500477k.
2. Humphrey, W.; Dalke, A.; Schulten, K. VMD: visual molecular dynamics. *Journal of molecular graphics* **1996**, *14*, 33-38.
3. Barrett, P.J.; Song, Y.; Van Horn, W.D.; Hustedt, E.J.; Schafer, J.M.; Hadziselimovic, A.; Beel, A.J.; Sanders, C.R. The amyloid precursor protein has a flexible transmembrane domain and binds cholesterol. *Science* **2012**, *336*, 1168-1171.
4. Beel, A.J.; Mobley, C.K.; Kim, H.J.; Tian, F.; Hadziselimovic, A.; Jap, B.; Prestegard, J.H.; Sanders, C.R. Structural studies of the transmembrane C-terminal domain of the amyloid precursor protein (APP): does APP function as a cholesterol sensor? *Biochemistry* **2008**, *47*, 9428-9446.
5. Beel, A.J.; Sakakura, M.; Barrett, P.J.; Sanders, C.R. Direct binding of cholesterol to the amyloid precursor protein: An important interaction in lipid-Alzheimer's disease relationships? *Biochimica et biophysica acta* **2010**, *1801*, 975-982.
6. Song, Y.; Hustedt, E.J.; Brandon, S.; Sanders, C.R. Competition between homodimerization and cholesterol binding to the C99 domain of the amyloid precursor protein. *Biochemistry* **2013**, *52*, 5051-5064, doi:10.1021/bi400735x.
7. Pantelopulos, G.A.; Straub, J.E.; Thirumalai, D.; Sugita, Y. Structure of APP-C99(1-99) and implications for role of extra-membrane domains in function and oligomerization. *Biochimica et biophysica acta. Biomembranes* **2018**, *1860*, 1698-1708, doi:10.1016/j.bbamem.2018.04.002.
8. Zhou, R.; Yang, G.; Guo, X.; Zhou, Q.; Lei, J.; Shi, Y. Recognition of the amyloid precursor protein by human γ-secretase. *Science* **2019**, *363*, doi:10.1126/science.aaw0930.
9. Bhattarai, A.; Devkota, S.; Do, H.N.; Wang, J.; Bhattarai, S.; Wolfe, M.S.; Miao, Y. Mechanism of Tripeptide Trimming of Amyloid β-Peptide 49 by γ-Secretase. *Journal of the American Chemical Society* **2022**, 10.1021/jacs.1c10533, doi:10.1021/jacs.1c10533.
10. Yang, G.; Zhou, R.; Guo, X.; Yan, C.; Lei, J.; Shi, Y. Structural basis of γ-secretase inhibition and modulation by small molecule drugs. *Cell* **2021**, *184*, 521-533 e514, doi:10.1016/j.cell.2020.11.049.
11. Svedružić Ž, M.; Vrbnjak, K.; Martinović, M.; Miletić, V. Structural Analysis of the Simultaneous Activation and Inhibition of γ-Secretase Activity in the Development of Drugs for Alzheimer's Disease. *Pharmaceutics* **2021**, *13*, doi:10.3390/pharmaceutics13040514.
12. Qi, Y.; Ingolfsson, H.I.; Cheng, X.; Lee, J.; Marrink, S.J.; Im, W. CHARMM-GUI Martini Maker for Coarse-Grained Simulations with the Martini Force Field. *J Chem Theory Comput* **2015**, *11*, 4486-4494, doi:10.1021/acs.jctc.5b00513.
13. Bhattarai, A.; Devkota, S.; Bhattarai, S.; Wolfe, M.S.; Miao, Y. Mechanisms of γ-Secretase Activation and Substrate Processing. *ACS central science* **2020**, *6*, 969-983, doi:10.1021/acscentsci.0c00296.
14. Liu, Y.; Zhang, W.; Li, L.; Salvador, L.A.; Chen, T.; Chen, W.; Felsenstein, K.M.; Ladd, T.B.; Price, A.R.; Golde, T.E., et al. Cyanobacterial peptides as a prototype for the design of potent β-secretase inhibitors and the development of selective chemical probes for other aspartic proteases. *Journal of medicinal chemistry* **2012**, *55*, 10749-10765, doi:10.1021/jm301630s.
15. Liu, L.; Ding, L.; Rovere, M.; Wolfe, M.S.; Selkoe, D.J. A cellular complex of BACE1 and γ-secretase sequentially generates Aβ from its full-length precursor. *The Journal of cell biology* **2019**, *218*, 644-663, doi:10.1083/jcb.201806205.
16. McDade, E.; Voytyuk, I.; Aisen, P.; Bateman, R.J.; Carrillo, M.C.; De Strooper, B.; Haass, C.; Reiman, E.M.; Sperling, R.; Tariot, P.N., et al. The case for low-level BACE1 inhibition for the prevention of

Alzheimer disease. *Nature reviews. Neurology* **2021**, *17*, 703-714, doi:10.1038/s41582-021-00545-1.
17. Aguayo-Ortiz, R.; Chávez-García, C.; Straub, J.E.; Dominguez, L. Characterizing the structural ensemble of γ-secretase using a multiscale molecular dynamics approach. *Chem Sci* **2017**, *8*, 5576-5584, doi:10.1039/c7sc00980a.
18. Bolduc, D.M.; Montagna, D.R.; Gu, Y.; Selkoe, D.J.; Wolfe, M.S. Nicastrin functions to sterically hinder γ-secretase-substrate interactions driven by substrate transmembrane domain. *Proceedings of the National Academy of Sciences of the United States of America* **2016**, *113*, E509-518, doi:10.1073/pnas.1512952113.
19. Lee, J.Y.; Feng, Z.; Xie, X.Q.; Bahar, I. Allosteric Modulation of Intact γ-Secretase Structural Dynamics. *Biophys J* **2017**, *113*, 2634-2649,doi:10.1016/j.bpj.2017.10.012.
20. Baker, N.A.; Sept, D.; Joseph, S.; Holst, M.J.; McCammon, J.A. Electrostatics of nanosystems: application to microtubules and the ribosome. *Proceedings of the National Academy of Sciences* **2001**, *98*, 10037-10041.
21. Krzemińska, A.; Moliner, V.; Świderek, K. Dynamic and Electrostatic Effects on the Reaction Catalyzed by HIV-1 Protease. *Journal of the American Chemical Society* **2016**, *138*, 16283-16298, doi:10.1021/jacs.6b06856.
22. Li, H.; Robertson, A.D.; Jensen, J.H. Very fast empirical prediction and rationalization of protein pKa values. *Proteins* **2005**, *61*, 704-721, doi:10.1002/prot.20660.
23. Li, Y.M.; Lai, M.T.; Xu, M.; Huang, Q.; DiMuzio-Mower, J.; Sardana, M.K.; Shi, X.P.; Yin, K.C.; Shafer, J.A.; Gardell, S.J. Presenilin 1 is linked with gamma-secretase activity in the detergent solubilized state. *Proceedings of the National Academy of Sciences of the United States of America* **2000**, *97*, 6138-6143.
24. Audagnotto, M.; Kengo Lorkowski, A.; Dal Peraro, M. Recruitment of the amyloid precursor protein by γ-secretase at the synaptic plasma membrane. *Biochem Biophys Res Commun* **2018**, *498*, 334-341, doi:10.1016/j.bbrc.2017.10.164.
25. Aguayo-Ortiz, R.; Straub, J.E.; Dominguez, L. Influence of membrane lipid composition on the structure and activity of γ-secretase. *Physical chemistry chemical physics : PCCP* **2018**, *20*, 27294-27304, doi:10.1039/c8cp04138e.
